# “…Keep mobile, I think that’s half the battle.” A qualitative study of prevention of knee pain in symptomless older adults

**DOI:** 10.1186/1471-2458-12-753

**Published:** 2012-09-07

**Authors:** Fizzah Ali, Clare Jinks, Bie Nio Ong

**Affiliations:** 1 Arthritis Research UK Primary Care Centre, Primary Care Sciences, Keele University, Keele, ST5 5BG, UK

## Abstract

**Background:**

The emphasis on prevention in English health policy continues to centre predominantly on major diseases such as coronary heart disease and diabetes. A number of key documents detailing self-management techniques and prevention of osteoarthritis (OA) are currently available, including the NICE guidelines and the Arthritis Foundation’s National Public Health Agenda for Osteoarthritis. However, few investigations have explored preventative knowledge of knee OA amongst the population. In particular, asymptomatic members of the population may use further information in considering how to prevent knee pain. This study considers perceptions around the prevention of knee pain amongst an asymptomatic population; this target population may provide alternative insights by which to stimulate preventative behaviours.

**Methods:**

A sample of thirteen patients with no current knee pain was selected from responders to a population survey. Each interview was tape recorded and fully transcribed. Qualitative computer software package NVivo8 was used to manage the data. Thematic analysis was conducted using the constant comparative method.

**Results:**

The definition and causes of knee pain were interpreted in a multitude of ways. The importance of prevention was recognised by a sub-set, while a small proportion of participants negated the role of prevention. A range of social factors, including early adoption of actions, influenced the implementation and continuation of preventative behaviours. Individual responsibility for prevention was a key theme, although the role of society was also considered. Exercise was cited as a principal preventative strategy, although some participants viewed exercise as a destructive activity. A number of participants deemed pharmacotherapy to be harmful and at odds with normal physiology, instead preferring to adopt preventative behaviour over medication usage.

**Conclusions:**

This asymptomatic population exhibit considerable breadth and variation in knowledge of preventative strategies for knee pain. Similarities in perceptions of prevention exist when comparing to the symptomatic population. These range from emphasis on individual responsibility, through to observations on the role of exercise and pharmacotherapy in knee pain. In general individuals are agreeable to act upon recommended treatments in line with NICE guidance. This receptiveness demands a greater consideration of preventative strategies in consultations, as well as wider availability and promotion of preventative strategies in order to improve the musculoskeletal health of the general population.

## Background

Musculoskeletal diseases are a prevalent public health problem with pervasive and enduring consequences; worldwide, an estimated 9.6% of men and 18.0% of women aged 60 and above have symptomatic OA [1]. Risk factors for knee OA have been identified [2], yet, little work has been carried out using this knowledge to shape prevention.

A number of publications are now available on the prevention of OA, for example, the National Public Health Agenda for Osteoarthritis 2010 [3] has listed several intervention strategies not only to control symptomatic OA, but additionally includes those with potential to prevent disease onset. Similarly, the National Institute of Health and Clinical Excellence (NICE) guidance “Osteoarthritis: The care and management of osteoarthritis in adults” illustrates the stepped key priorities for implementation in patients with OA [4].

Individuals in the community practice a variety of self-management techniques to manage their OA, some are in accordance with the NICE guidelines and others are not [5]. However, there remains a distinct dearth of studies exploring preventative knowledge of knee OA amongst the general population. More specifically, asymptomatic members of the population with or without previous experience of knee pain may provide an alternative angle by which to consider the prevention of knee pain. Such symptomless adults may generate useful information about beliefs among the general population, permitting wider and contextual indications for stimulating preventative behaviour in knee OA.

In this paper we will report on a study examining the perceptions on prevention of knee OA amongst symptomless adults. We will outline the study design, followed by a thematic presentation of the findings, and draw together the conclusions in the discussion section.

## Methods

This study aims: first, to explore individuals’ perceptions of knee pain and their notions of the course and severity of the condition. Second, to investigate how the concept of prevention of knee pain is understood, interpreted and applied. Third, to examine whether and if so, how symptomless older adults translate their preventative knowledge into specific behaviour, including an understanding of drivers or barriers to implementation. Finally, present the results of this analysis to a multi-disciplinary group of health professionals in order to discuss the implementation of preventative strategies into health practice.

With the main aim being to understand people’s beliefs, interpretation of and attitudes to prevention, a qualitative study design was considered most appropriate - using semi-structured interviews as the key data collection method. The interviews asked people to tell their own ‘health story’ before focusing on knee pain, using the Dahlgren and Whitehead model of social determinants of health [6].

A purposive sample of people with and without knee pain in the last 12 months was drawn from an on-going cohort study of joint pain in the local population. We wrote to 180 people who had given consent to further contact and invited them to participate in an interview about views of prevention of knee pain. We purposively invited people who reported no pain in the survey but who had at least two risk factors (e.g. a previous knee injury, a body mass index of 25 or over and classified as overweight or obese, or had a previous manual job). We also selected an equal number of men or women and sampled to ensure people were invited from across three age groups (50–64, 65–74 and 75 years and over).

Our sample of thirteen participants is drawn from the total of 28 people who replied to the interview invitation and included four females and nine males. All had reported no knee pain in the survey and were aged between 59 and 86 years old. The larger study (including those with knee pain is reported elsewhere [7]). Each interview was tape recorded and fully transcribed, lasting between 40 and 90 min. All interviews were conducted by a single interviewer. Common information and consent procedures were adopted in line with the approval by the Local Research Ethics Committee.

Three researchers read the 13 transcripts independently. Two researchers had been involved in the other part of the study (e.g. exploring views of people with knee pain) and one researcher was ‘naive’ i.e. new to this study and new to the data. Using a naive researcher has been described by May and colleagues as an important strategy for secondary analysis to avoid confirmatory explanation and allow for critical and exploratory analysis [8]. While this study is not strictly a secondary analysis, the lack of prior knowledge of the naive researcher did allow a fresh look at the whole transcripts and the new focus on the accounts of the participants without current knee pain to be ‘uncontaminated’ by earlier analysis. A coding framework was developed through an iterative process by the naive researcher, and the final coding framework was then produced through discussion and comparison of data between the three researchers and this was used to code all transcripts. The qualitative computer software package Nvivo 8 was used to systematically organise the data. Analysis of the coding groups permitted ordering of concepts and exploration of links and relationships between recurrent themes. Memos were also written in order to aid development of ideas, allowing early comparisons and routes to pursue. The principal themes to emerge from the data are outlined below.

## Results

### Perceptions of knee pain

#### Defining knee pain

Eight participants talked explicitly about their interpretation of knee pain. They differentiated between knee pain and a tolerable discomfort, ache, twinge or stiffness, which some of them had experienced, and used phrases such as:

‘…sort of began to have niggly discomfort. Erm, and I’m not saying I had knee pain because I didn’t. It was just discomfort and stiffening, a little bit.’ (0968)

The notion of knee pain was defined as having a greater impact such as an unpleasant sensation in the knee above a particular threshold and affecting daily life. The degree of disability generated by knee pain was inextricably tied with perceived effect on lifestyle, which varied considerably:

‘I used to know quite a few of, erm, his friends and I used to go (to) the races and things like that. You’d be surprised how many have dropped out because of, like, knees or hips and things like that.’ (6900)

For some individuals arthritic symptoms were perceived as ‘normal’:

‘If you stopped (for) every little aches and pain (that) you had, you’d never do nothing, would you? And it’s never…never stops me (from) doing anything, does it, like?’ (02)

Knee pain was perceived as a diverse phenomenon and sometimes considered as transient, episodic and self-limiting.

‘You do get a little bit of stiffness, sometimes but after an hour or two walking about, it goes.’ (0968)

Knee pain, in accordance with other chronic pain, was considered a dynamic entity varying over time. For example, one participant regarded knee pain as a recurrent phenomenon, and when describing his wife’s suffering, he stated:

‘Oh, she’s had a bit of ‘run’ more recently – bit of a flare-up on her knee, yeah. She’s happened…these last few weeks, actually. I think it’s easing off a bit, now’. (6150)

The above accounts show knee pain is defined in a variety of ways, ranging from a normal phenomenon, an episodic self-limiting stiffness to an unpleasant sensation that affects daily life. Thus, it is a dynamic concept shaped by personal contexts. This impacts on whether or not an individual classifies themselves as in pain or indeed symptom free.

#### The causes of knee pain

Perceptions such as ‘old age’ and ‘wear and tear’ are frequently cited as causative attributions [9], and such findings can be extended to the perceptions held by the asymptomatic population described in this study. The connection between pain as a ‘normal’ part of the aging process has previously been reported [10], and was frequently alluded to by participants in this study. The notion of an inevitable and progressive breakdown of the musculoskeletal system was reinforced through the idea that a number of similarly aged individuals face the same trouble.

‘…we’re a group of… all of a similar age and we’ve had a ‘pretty’ bad time with the …bodies breaking down so often.’ (0957)

One interviewee, however, did make reference to the possible insignificance of age in relation to joint replacements:

‘I’ve loads of friends that’ve had replacement joints in the knees and not old people…not all old people, you know – some younger people and they can have them scraped, can’t they? – sometimes they…they make a slit and scrape the cartilage or whatever it is and that seems to work for some people…’ (0957)

The notion of impact and pressure on knees produced by obesity and thus generating knee pain was recurrent. The knees were perceived as shock absorbers, on which the application of excessive pressure produced damaging consequences.

‘…there must be a connection because you…the more weight you put on them, there must you know, like with that…with feet. I mean…what did I say…your legs aren’t made to carry excess weight, are they?’ (6900)

The importance of improving the strength of surrounding muscle to prevent knee pain was alluded to and the ‘pressure concept’ affected the implementation of preventative behaviour, such as exercise. For instance, the notion that the lower limb is not designed to bear excessive weight fed the perceived importance of weight reduction proportional to implementation of exercise:

‘[exercise can do] more damage than good, in some cases can’t it? Make sure you’re all right. Exercise and I suppose, if you particularly stiff and that…but…try to lose a bit of weight so you aren’t putting pressure on your limbs…’ (6900)

A variety of responses indicated causative factors to range from over-use and excess pressure, through to degenerative and inevitable mechanisms. However, some individuals were unable to cite reasons for their knee-pain free existence. This included reference to ‘luck’ as well as inherited factors. Taken together these ideas produced the impression of individual powerlessness against preventing knee pain. Yet, this was variable where one respondent believed in the role of preventative moderation, aided by the element of ‘luck’:

‘if you exercise all your life, you know, in moderation, I think it keeps your joints going, but, you know…other than, er, if you’re just unlucky, ‘cos you can have…I suppose you could have a set of twins and one twin might…might be more fortunate than the other. There’s no ‘hard and fast’ rule, is there? But, it certainly does help if you do exercise from an early age.’ (02)

Many participants reflected beliefs that have been reported in the literature, linking joint pain with growing older. Some talked about prevention in terms of improving muscle strength, controlling weight and moderate exercise, while others combined these factors that are outside one’s control mentioning luck and genetic make-up.

#### The importance of knee pain

The prevention of knee pain was deemed important as its consequences generated limitations. One participant offered a particularly rich discourse detailing his understanding of the day-to-day impact of knee pain through his observation of his father’s condition. While physiotherapy alleviated the knee pain, it did not sort his overall bodily pain:

‘…my father was alive and he was crippled with, erm, knee joint…with joint problems…he was in considerable pain and discomfort…I asked this physio if he would have a look at my father…within the space of a couple of treatments, he straightened his legs before my very eyes…but the problem was getting my father from A to B and the journey – the movement of the vehicle – and because of his arthritis…that it knocked him up…the benefit he was getting to his knees, er, was, you know, counterbalanced by his bodily pain…’ (6028)

The participant became familiar with the impact of joint problems through his father’s disease and used this as a learning experience to change his own health-seeking behaviour; using regular and immediate physiotherapy if muscular discomfort occurred, thereby preventing a deterioration in locomotor function:

‘…I did learn a big lesson from that…the moment I have any of this pain, I tell him and he works on that shoulder and, erm, I can now play my accordion for as long as I like…I do feel that I learnt, late in life, that what we should be doing is, er, following a…a regime…or whatever, er…preventative and not wait until we’ve got a critical condition and then need to reverse it.’ (6028)

In relation to her back-pain one participant similarly mentions discomfort and limitations in movement:

‘…if I don’t get some help with it…I’ve only got three weeks before my holiday and I don’t want to be, you know extra slow, extra slow moving around…plus the discomfort of it…’ (0957)

Previous studies have highlighted a dynamic chronic disease hierarchy with unpredictable or uncontrolled conditions [11] alongside life-threatening diseases causing most concern. This was re-iterated in our study where individuals prioritised conditions such as myocardial infarction over knee pain, and changes were made to adopt a healthier lifestyle or employ additional preventative measures. Interestingly, the participants also prioritised problems within the realm of musculoskeletal disease:

‘a back…it controls everything doesn’t it really? Your back muscles, you know, you…if you’ve got a good back, you can almost do anything. If you haven’t got a good back, you can do little – you’re…it restricts you terribly, you know. I…well, all joints would do, but back is…it’s a main…a lot of muscles in your back. You depend on them for walking; you depend on them for everything – mobility…and if your back is bad…I know a lot of people don’t realise that the back…the importance of it…’ (3641)

Participants noted physical discomfort and ensuing limitation of movement as disruptive. Previous experience of adverse health events was sometimes used as inspiration to change behaviour and implement preventative activities. Although joint pain at large was seen to generate uncomfortable symptoms, people prioritised their health problems even within the realm of musculoskeletal diseases.

### Prevention of knee pain

#### Can knee pain be prevented?

In our study attitudes to knee pain as a preventable condition varied, ranging from those individuals who thought knee pain could be avoided through appropriate measures, through to those respondents who regarded knee pain as preventable but were unable to explain how, and finally those who denied that prevention was possible. Some participants in the first group saw knee pain as a path along which individuals ‘slipped down’, and timely prevention was considered key to stop advance into a territory wherein prevention was no longer beneficial or whereby the individual had evolved into the state of an ‘invalid’:

‘I think you reach a point where you’ve got to…you’ve got to be able to take action to try and, you know, stem it.’ (0957)

Preventative measures, including exercise, the use of Cod Liver Oil, Chondroitin and Glucosamine tablets, weight loss, use of joint supports such as Tubigrip, appropriate posturing as well physiotherapy were mentioned as evidence by respondents to explain their continuing asymptomatic nature. Two respondents cited ‘common sense’ in association with prevention generally: the combination of a healthy diet, exercise and accident avoidance were all considered as ‘obvious’ factors in preventing ill-health.

Four respondents fell in the second group who were unsure whether knee pain was preventable, with one of the respondents saying:

‘…no, I’ve never given it a thought, you know. In other words, I…if I ignore it, it won’t come, you know. No, I’ve never thought about it, no.’ (6900)

Another participant believed knee pain to be a preventable entity but was unable to specify what prevention entailed:

‘I didn’t do anything special about my knees at all, you know – just kept going, and, er…and I was lucky because, erm, you know, whatever I did, did obviously seem to respond pretty well to…you know, so…’ (0957)

Finally, the people who did not think prevention was possible saw knee pain as inevitable, or felt unsure about the impact of contextual factors:

‘I don’t know, because I don’t know the circumstances of people that have…that have got it’. (4237)

One individual constructed a barrier around his individual social circumstances generating his own ‘health level’. He provided a rationale whereby he reasoned that people could expect a certain lifespan, and living beyond that was a question of ‘luck’ and not within his own control. Thus, preventive behaviour was not seen to be relevant:

‘…everybody’s allocated three score and ten – seventy years old. So, I’ve reached that now, so I’m on…I’m on a bonus, now, aren’t I?’ (6150)

Given the range of ideas expressed in our study a single approach to preventing knee OA is clearly not appropriate. There were great variations in participants’ perceptions of whether knee pain could be prevented. While some believed that knee pain was inevitable, there were participants who cited preventive measures like exercises, weight reduction, physical support and health supplements like Cod Liver Oil, Chondroitin and Glucosamine as their reasons for not having knee pain.

#### Implementing preventative behaviours

A number of participants talked about implementing prevention into their life, for example through exchanging car use for short distance travel with walking instead:

‘if it’s only walking ‘down’ to the main road to the Post Office, you know. I can…I can do that. I’ll do…if I’ve just got something to post, I won’t get the car out, you know. I will walk it and I’ll try and walk as quickly as possible and then it makes it a bit more…a bit more ‘worth while’, you know.’ (0957)

The importance of a regular routine incorporating preventative measures, a supportive family network and deriving noticeable reward from implementing preventative behaviour spurred continuation of the activity:

‘it’s helped her control her weight and I think she’s lost it because her tummy’s gone down and…her knees are beginning to look more shapely, you know, and I think she’s aware of this, now. So I think that is a little spur…’ (0968)

Enjoyment of a preventative activity, such as exercise, allowed easier execution in daily life and was primary to the beneficial health effects of the behaviour:

‘No, it comes…it just comes natural(ly), like…you know, it…don’t…I never stop and think, ‘ooh, this is doing me great…yeah, this is doing me good.’ I just do it then hope it’s doing me good, yes…and…and…’touch wood’, I’ve had no problems at all, so…’ (6900)

The early adoption of preventative activities was perceived as a significant factor in continuing activities later on in life. Nine respondents discussed taking up exercise early in life, whether as gym membership, competitive sports or as part of occupation or having a healthy diet.

‘I think, in general, the most important thing is to try and keep active and from an early age…’ (4237)

On the other hand, a variety of factors also posed as barriers to implementing preventative measures. With regards to exercise, the presence of other musculoskeletal problems and physical exhaustion hindered:

‘I do play tennis and I probably play tennis too long with this arm. Once it became noticeable, I stopped, but it’s a very specific area.’ (0957)

Social and environmental factors played a role. The price of gym membership as opposed to a free ten week gym course on prescription; lack of peer group; proximity of leisure facilities; and both weight and age were described as factors limiting self-esteem and thus desire to attend the gym:

‘…when you’re overweight and over age, yes, darling, I do. I mean when you’re young, you’re confident and you can bluff your way in everywhere, but when you’re older, you’re, er…sort of, more…shyer, really, you know, and thinking, ‘I can’t wear tights; I can’t wear leotards,’ you know. So, I just wear black training-trousers and a black tee-shirt and feel quite good…” (0968)

For one respondent the illness of her spouse, i.e. her role as a carer impacted on her ability to make time for preventative activities:

‘I mean, I did keep up the tennis playing, most of the time, because I’d just be away about an hour and a half and…I mean, he wasn’t absolutely, you know, totally immobile. He could manage in the house okay, but, erm, you know, I didn’t like being out too long.’ (0957)

Finally, environmental factors such as fast-food availability and cost of healthier food were seen as barriers to preventative behaviours.

#### Responsibility in prevention

Responsibility for preventing knee pain through implementing precautionary strategies was a recurrent notion. The majority of respondents attributed responsibility to the individual, including motivation to both initiate and persist with health promoting activities, and often smoking cessation was used as an example.

Three participants regarded family members as important influences in the early adoption of preventative behaviour, with parents being role models and inculcating key preventative behaviours that people continue into adulthood:

‘I think at that stage, really, it’s in the hands of whoever’s running the household – whether the mother or father or…[…] you know instilled good habits […] I mean both sons, now…they’re both… they both take plenty of exercise…different types, but, you know, they do and I think, ‘well, maybe if they hadn’t have had quite so much when they were young or been encouraged when they were young, maybe they wouldn’t have done it.” (0957)

The responsibility of health professionals and the NHS generally was outlined by one participant:

‘I think the system, medically, everyone ought to go (for tests) at aged 60 […] if everybody at 60 went for an MoT, to check their blood-pressure, my condition would’ve…started to surface, you know.’ (5456)

For those individuals who are not motivated to implement preventative behaviour, ‘the state’ was cited as important in playing a role in providing a ‘kick-start’, for example with the initiation of small exercise programmes on a local basis at residential areas.

‘I suppose, in the first instance, it’s the individual, but people, being individuals, are all so different, so I think that there should be some sort of, ‘gee-up’ from authorities to… I mean, for instance, a place like this. It might be done – I don’t know – somebody could come round from the Council or some gym or something…just do a few basic exercises with the residents…’ (6900)

Conversely, in instances Government policy and guideline was seen to lack influence and thus disregarded:

‘I really don’t know how you will get through to some people. I really don’t.’ (0968)

Individual responsibility for prevention was cited as the most important factor, but some participants could see a role for professionals, the NHS or the government in order to influence the population generally.

#### Influential sources

Health information was obtained from a range of sources, including from celebrities, articles, adverts and television. Sources such as family members, health professionals, and others including exercise trainers were also mentioned. All 13 participants referred to the importance of peer group influence in terms of inspiration or stimulating engagement in activity, or providing support to be involved with preventative behaviours:

‘…once I’d been a couple of times, erm, I thought, ‘oh yeah. They’re all the same as me. They’re all over weight and, er, they’ve all got health problems.’ So it…yeah, it was great for me and it still is.’ (0957)

In one instance, the peer group was used as a vehicle to inform and encourage healthy behaviours in others:

‘…we said to her, you know, if you’re not careful, you’re going to be dead in another five years and after that she started at weight-watchers…’ (6598)

Most participants seemed to have gathered information opportunistically. This information was noted to be important to initiate preventative behaviours. For example, regarding non-specific health promoting measures, one participant altered his dietary choices:

“I read an article about, erm, pork and bacon production in the EEC and I thought, ‘right, after reading that, I’m not going to buy any bacon or pork that is produced in the, erm, EEC…I do check when I buy bacon that it is UK produced, where from what I can gather, the conditions are, you know, better…” (0957)

Participants were explicitly aware of the importance of health education with statements such as “I think people need to have a bit more awareness” (6028); “…certainly we could do with education over… on health” (6028) and “…had I had a…more of an awareness, then maybe I could have avoided or delayed, you know what befell me…” (6028). It was further noted that information had to be relayed in an appropriate manner: “The average ‘thick-o’ does not read many papers, but they’re glued to the telly” (0968).

#### Exercise and knee pain

Exercise was the most commonly cited factor implicated in disease prevention:

‘As I say, if they get plenty of exercise and, er, keep mobile, I think that’s ‘half the battle’…’ (5456)

Respondents varied in their definition of ‘exercise’, for some it constituted activities offered by gymnasiums such as dancing, swimming, ‘the rowing machine’, ‘the cycling machines’ or ‘walking machines’. For others ‘exercise’ was considered in a low key manner, considered as simply ‘activity’ or ‘physical things’ such as that encompassing walks, household activities including gardening and occupation. Specific movements tailored to joint problems advised by a physiotherapist or a chiropractor were also considered as exercise. Non-exerting forms of activity such as knitting were considered as helpful to specific parts of the body, aiding in preventing ‘rheumatism in their hands’ and keeping ‘your hands going’. The perceived importance of exercise is summarised by one interviewee: ‘keep moving every day’.

Exercise was generally presented as ‘constructive’, but also as having the potential to be ‘destructive’ if done excessively, with many respondents favouring ‘moderation’. All thirteen participants viewed exercise as beneficial. One respondent cited the importance of exercise in preventing her knee pain:

‘D’you think if you didn’t go to the gym…d’you think your knees would’ve got bad? Oh, I think they might have done, yes…’ (0968)

Walking was also directly cited as beneficial for knee pain:

‘At the end of the year, the walking really loosened-up my knees and I couldn’t believe it…’cos I was quite tired with all the walking, but yet my knees were okay.’ (0957)

Toning up and keeping your muscles ‘in better shape’ was perceived to keep joints ‘in better shape’, as well as maintaining ‘mobility’ and thereby ensuring muscles and joints function satisfactorily. Phrases such as ‘if you don’t use it you loose it, kid’ and ‘anything seizes-up if you don’t use it…’ were recurrent and tied in with the notion of joint flexibility generated through use.

Exercise was also noted to be beneficial in terms of both weight loss and weight control, which in turn was connected with knee pain and wider health problems:

‘Now, it doesn’t make me lose weight, but it helps me control my weight and I think if my weight is controlled, I don’t have any, er, circulation or knee trouble.’ (0957)

Aside from recognition of direct physical benefits, the social aspects of exercise including the side-effect of establishing a social network, ‘company’ and ‘feel(ing) good’ was raised. Moreover, the physical effect was held in close association with the mental effects:

‘…if you’re physically active, you’re mentally active as well, ‘cos you’re thinking things, aren’t you? – in a way you aren’t just stagnating, are you?’ (6900)

The enjoyment of exercise featured as an integral notion to the up-keep of exercise. For some the enjoyment came as a primary factor for carrying out exercise, with the beneficial health effects cited as secondary. Where exercise was seen to be boring, for example, in the gym setting it was continued because of its merits in providing an opportunity to conduct exercise that would otherwise not be done regularly.

The benefits of exercise to prevent future knee pain were seen by some to be enhanced through supplementation with other actions:

‘I just didn’t want to go ‘down that path’, so I…I tried to prevent it, you know, by going to the gym and, er, taking Cod Liver Oil tablets…’. (0968)

Six respondents considered exercise as a potentially ‘destructive’ activity, believing that too much exercise had the ability to either elicit or aggravate musculoskeletal pain. Pushing the body by, for example, weightlifting was seen as damaging cartilage and muscles. Physical exhaustion was also described as a limiting factor. Dancing was cited as causing knee problems, through affecting ligaments, although in this instance resulting knee ‘stiffness’ is tolerable:

‘…you can get a little bit of knee trouble – not trouble, really, but, you know- you’re doing the tight turns and twists and things they do. You do get a little bit of stiffness, sometimes, but after an hour or two, walking about it goes. So, its just a general muscle extension – or probably a pull of a ligament. I don’t know. But its nothing that troubles me, you know, in any great way.’ (3641)

Ten participants described exercise in ‘moderate’ terms and thus defining it as being beneficial. The notion of ‘moderation’ was used in conjunction with the idea of doing as much exercise as the human body permitted, or as much as was seen to be ‘natural’. The idea of carrying out a reasonable amount of exercise was often tied to age:

‘…by the time you get to my age, which is, sort of, mid…later 60s…you’ve got …you’ve got to be reasonable about it.’ (0957)

This was linked to the idea of knowing one’s own limit and thereby permitting continuation of activity. It was important to do exercise within perceived capabilities:

‘I feel I want to be able to exercise, absolutely as long as I can do and I feel that I’m better if I don’t ‘push it’ too much, so that I can keep, you know…keep going’. (0957)

The human body was perceived as able to direct the individual into understanding what constitutes too much activity for them, and that ‘listening’ to their bodies and ‘slowing down’ was important. These ideas tied in with the notion of the supposed healing properties of ‘Nature’:

‘That has got to be put right, by nature in its own time and in its own way. You’ve got to help it by not over-doing it; not stretching it; just let it do it – work itself, like they can do.’ (3641)

One respondent discussed the ‘natural’ role of exercise in a role other than preventative:

‘…animals and men and women are born to be active. They’re not born to be vegetables and lie about, but they…so, Nature’s telling you, ‘move about; if you’re feeling an injury, don’t lie on it and take drugs’. (3641)

The perceptions about exercise did go beyond pure physical and area-specific benefits, and included social and psychological elements. Furthermore, individual differences and the role of nature shaped many people’s thinking.

#### Pharmacotherapy in knee pain

Respondents were aware of a number of forms of medication available for alleviation of knee pain. The idea of using medication for joint pain was viewed as a second resort, instead permitting ‘nature’ the first opportunity to repair. In fact, the idea of possibly having to take medication was used as motivation to implement preventative activity:

‘So I thought…I never had to have medication, but I… that’s when I thought, ‘right, A. (female first name) you’ve got to do something about this!’ (0957)

Steroid injections, analgesics in association with rest, heat treatment were noted as beneficial for musculoskeletal pain. The potential benefits of Cod Liver Oil tablets were noted and individuals adjusted the doses of this as they felt appropriate, titrating to the sensations in their knees. Cod Liver Oil was also used as a preventative measure:

‘I didn’t want to ‘go along that path’ so I tried to prevent… I started about six years ago, by starting (going to) the gym and starting the Cod Liver Oil. Now whether they both helped me is… that’s just my opinion and I think (that) they did.’ (0968)

In many responses medications were presented as ‘un-natural’, potentially containing hidden ‘residue’, polluting the ‘blood-stream’ or the human body. Additionally, analgesics were perceived to ‘mask’ pain generating the false perception of improvement in conditions with resultant return of the symptoms as ‘the effects of the drug are wearing off’. A more reasonable approach was considered to involve resting the joint or allowing a ‘natural’ healing process to occur over time followed up by alternative health measures, if necessary:

‘I think people look…fly to…the first thing is to a pill is nonsense. Give it time, first. If you get an injury, give it time; give it a couple of days – three days or maybe four days. You may feel it’s not improved (and) if it doesn’t improve, then you think, well you’ll have to do something else.’ (3641)

Pharmacotherapy was perceived to be at odds with the ‘Nature’. Today’s generation were described as ‘hypochondriacs’; seeking help from medical professionals and using a ‘load of pills’. The idea that such medication was beneficial was tempered with doubt:

‘…they think, ‘ooh! that’s…they did me good,’ but it might not have been that. It might have been the nature…Nature’s way of curing. It may have gone right on its own. So you’re never sure, you see, if you take a load of pills, you’re never sure whether it’s the pill or what.’ (3641)

Most participants expressed their reluctance to take pain medication and saw preventive action as a strategy to avoid medicines. Again, the notion of nature was often cited and giving the body an opportunity to heal itself.

## Discussion

This study sought to understand ideas around the prevention of knee pain in a group of asymptomatic adults. Most studies focus on people with symptoms, but by selecting this group we intended to explore perceptions about prevention that are not directly influenced by current pain or treatment, thus accessing thinking of a general rather than a clinically defined population.

In order to tackle musculoskeletal disorders as a public health problem it is necessary to understand the lay population’s understanding of the entity. In this instance, ‘knee pain’ is defined in various ways: through notions of intensity and impact on life; character and chronicity, as well as duration. The interpretation of knee pain as a ‘normal’ phenomenon may inhibit people from considering its prevention. This notion may be linked with the previously documented perception, and confirmed by this study, of knee pain as an inevitable and incurable outcome of an aging process [12,13]. Three schools of thought were revealed in our study with regards to the prevention of knee pain. First, those individuals who believe knee pain can be prevented; second, those who believe in prevention but are unsure what it entails and third, a group that dissociates prevention from knee pain. The latter two groups should be the targets for public health messages. Prevention may be encouraged through highlighting the potential long-term and wide-ranging impact of knee pain on one’s quality of life, and the benefits of timely preventative action.

Our study population demonstrated a breadth of preventative knowledge, including the use of exercise and pharmacotherapy. Similar to symptomatic individuals, our cohort perceived physical activity to promote health gain, provide symptomatic relief and generate a sense of well-being [14]. The health benefits of physical activity included joint-specific effects such as improving muscular strength, joint flexibility as well as general effects like weight reduction and control. Reduction in pain and improved mobility featured as principal improvements in symptoms. However, participants also considered exercise as potentially injurious, with fears of subsequent aggravation of disease through mechanical injury, potential reduction in function and exacerbation of pain. Symptomatic populations also display variable opinions on the benefits of exercise [15,16] with some individuals reporting increased pain with exercise [14]. Holden et al. [16] found considerable uncertainty about the role of exercise in managing knee symptoms. Barriers and facilitators to exercise and physical activity included those relating to the person (e.g. enjoyment), symptoms (e.g. pain) and social and environmental factors (e.g. lack of facilities, weather). The importance of understanding the limits to exercise has therefore been highlighted in both groups.

While our participants recognised the general benefits of physical activity, they showed a reluctance in taking painkillers, and cited analgesics as being ‘un-natural’ and disruptive of the body’s inborn healing mechanisms. Resistance to taking medication has been documented in other studies [17][18], while willingness to take ‘alternative’ preparations to analgesics has been recorded [19]. Participants in our study did not have knee pain, but were equally reluctant as symptomatic OA patients to accept pain-relieving treatment [20].

Notions of prevention of knee pain were broadly in line with evidence, and people actively sought out information and ‘ratification’ by peers. Like symptomatic adults prevention was largely seen as an individual responsibility [7] even though social and environmental factors were recognised as potentially facilitative.

Returning to the original aim of exploring the perceptions of asymptomatic individuals, the study has highlighted more similarities than differences with people who currently suffer with knee pain. It appears that knowledge about the importance of physical activity has permeated into the general population, and that the two groups only differ in terms of emphasis. A belief in maintaining levels of activity, and integrating these wherever possible into daily routines was stronger in the asymptomatic group. This may be because knee pain is in the background rather than in the foreground [21] and being physically active is an actual and positive reality. In terms of the implications of our findings for clinical practice, the use of the NICE OA guidelines in consultations for knee pain appears to fit with lay experiences and those who are symptom free. However GPs and other primary care professionals may not be sufficiently familiar with this evidence and it is important that this is remedied because people consulting for knee pain are open to acting upon many of the recommended treatments.

## Conclusions

Our study has provided insight into the asymptomatic individual’s knowledge and practices in order to avoid potential OA related knee problems, where lifestyle factors play an integral role. English health policy on prevention continues to focus primarily on major diseases, but a further drive to implement mutually beneficial preventative strategies i.e. those shared by both musculoskeletal conditions and higher profile disease states, could potentially lead to improved musculoskeletal health in a wider section of the population.

## Competing interests

The authors declared that they have no competing interest.

## Authors’ contributions

CJ and BNO conceived the design of the study. Data interpretation was carried out by FA, with CJ and BNO input. All authors contributed to the development of the manuscript. All authors have read and approved the final manuscript.

## Pre-publication history

The pre-publication history for this paper can be accessed here:

http://www.biomedcentral.com/1471-2458/12/753/prepub
